# Supplementary material for: Pyruvate Dehydrogenase Kinase 1 inhibition mediated oxidative phosphorylation enhancement in cartilage promotes osteoarthritis progression
Source: BMC Musculoskelet Disord. 2023 Jul 20;24:597. doi: 10.1186/s12891-023-06585-6 (PMC10357736; doi:10.1186/s12891-023-06585-6)
Supplement: Supplementary file 1 — Additional file 1. [file 12891_2023_6585_MOESM1_ESM.zip › MF.Gsea.1653627316242/GOMF_POLY_PURINE_TRACT_BINDING.html]

Details for gene set GOMF\_POLY\_PURINE\_TRACT\_BINDING[GSEA]

|  || Dataset | 55457\_collapsed\_to\_symbols.cls.cls #OA\_versus\_normal.cls.cls #OA\_versus\_normal\_repos |
| Phenotype | cls.cls#OA\_versus\_normal\_repos |
| Upregulated in class | normal |
| GeneSet | GOMF\_POLY\_PURINE\_TRACT\_BINDING |
| Enrichment Score (ES) | -0.4392737 |
| Normalized Enrichment Score (NES) | -1.4013499 |
| Nominal p-value | 0.08828523 |
| FDR q-value | 0.2616454 |
| FWER p-Value | 1.0 |
Table: GSEA Results Summary

  

Fig 1: Enrichment plot: GOMF\_POLY\_PURINE\_TRACT\_BINDING      
 Profile of the Running ES Score & Positions of GeneSet Members on the Rank Ordered List

  

| SYMBOL | TITLE | RANK IN GENE LIST | RANK METRIC SCORE | RUNNING ES | CORE ENRICHMENT || 1 | PPIE | peptidylprolyl isomerase E [Source:HGNC Symbol;Acc:HGNC:9258] | 334 | 0.733 | 0.0600 | No |
| 2 | ATXN1 | ataxin 1 [Source:HGNC Symbol;Acc:HGNC:10548] | 2862 | 0.264 | -0.1052 | No |
| 3 | DDX1 | DEAD-box helicase 1 [Source:HGNC Symbol;Acc:HGNC:2734] | 3305 | 0.221 | -0.1137 | No |
| 4 | KHDRBS1 | "KH RNA binding domain containing, signal transduction associated 1 [Source:HGNC Symbol;Acc:HGNC:18116]" | 3748 | 0.182 | -0.1267 | No |
| 5 | MCRS1 | microspherule protein 1 [Source:HGNC Symbol;Acc:HGNC:6960] | 4286 | 0.129 | -0.1533 | No |
| 6 | KHDRBS2 | "KH RNA binding domain containing, signal transduction associated 2 [Source:HGNC Symbol;Acc:HGNC:18114]" | 4550 | 0.109 | -0.1609 | No |
| 7 | ZC3H14 | zinc finger CCCH-type containing 14 [Source:HGNC Symbol;Acc:HGNC:20509] | 4896 | 0.080 | -0.1783 | No |
| 8 | RBMS1 | RNA binding motif single stranded interacting protein 1 [Source:HGNC Symbol;Acc:HGNC:9907] | 7121 | -0.090 | -0.3405 | No |
| 9 | EIF4A3 | eukaryotic translation initiation factor 4A3 [Source:HGNC Symbol;Acc:HGNC:18683] | 7646 | -0.130 | -0.3659 | No |
| 10 | FMR1 | FMRP translational regulator 1 [Source:HGNC Symbol;Acc:HGNC:3775] | 7944 | -0.151 | -0.3712 | No |
| 11 | RBMS2 | RNA binding motif single stranded interacting protein 2 [Source:HGNC Symbol;Acc:HGNC:9909] | 8373 | -0.184 | -0.3829 | No |
| 12 | RBPMS | "RNA binding protein, mRNA processing factor [Source:HGNC Symbol;Acc:HGNC:19097]" | 8547 | -0.197 | -0.3733 | No |
| 13 | PABPC4 | poly(A) binding protein cytoplasmic 4 [Source:HGNC Symbol;Acc:HGNC:8557] | 8741 | -0.212 | -0.3634 | No |
| 14 | RBMS3 | RNA binding motif single stranded interacting protein 3 [Source:HGNC Symbol;Acc:HGNC:13427] | 8981 | -0.230 | -0.3550 | No |
| 15 | HNRNPU | heterogeneous nuclear ribonucleoprotein U [Source:HGNC Symbol;Acc:HGNC:5048] | 9847 | -0.306 | -0.3863 | No |
| 16 | ELAVL4 | ELAV like RNA binding protein 4 [Source:HGNC Symbol;Acc:HGNC:3315] | 10412 | -0.362 | -0.3876 | Yes |
| 17 | TIA1 | TIA1 cytotoxic granule associated RNA binding protein [Source:HGNC Symbol;Acc:HGNC:11802] | 10855 | -0.411 | -0.3737 | Yes |
| 18 | LARP4 | La ribonucleoprotein 4 [Source:HGNC Symbol;Acc:HGNC:24320] | 11700 | -0.533 | -0.3768 | Yes |
| 19 | PABPC3 | poly(A) binding protein cytoplasmic 3 [Source:HGNC Symbol;Acc:HGNC:8556] | 12257 | -0.663 | -0.3422 | Yes |
| 20 | HNRNPDL | heterogeneous nuclear ribonucleoprotein D like [Source:HGNC Symbol;Acc:HGNC:5037] | 12522 | -0.759 | -0.2736 | Yes |
| 21 | PABPC1 | poly(A) binding protein cytoplasmic 1 [Source:HGNC Symbol;Acc:HGNC:8554] | 12562 | -0.778 | -0.1854 | Yes |
| 22 | DAZAP1 | DAZ associated protein 1 [Source:HGNC Symbol;Acc:HGNC:2683] | 12686 | -0.854 | -0.0948 | Yes |
| 23 | DDX3X | DEAD-box helicase 3 X-linked [Source:HGNC Symbol;Acc:HGNC:2745] | 12788 | -0.949 | 0.0087 | Yes |
Table: GSEA details [plain text format]

  

Fig 2: GOMF\_POLY\_PURINE\_TRACT\_BINDING      
 Blue-Pink O' Gram in the Space of the Analyzed GeneSet

  

Fig 3: GOMF\_POLY\_PURINE\_TRACT\_BINDING: Random ES distribution      
 Gene set null distribution of ES for **GOMF\_POLY\_PURINE\_TRACT\_BINDING**
